# Observations on Hereditary Hemorrhage

**Published:** 1828-10

**Authors:** Reynell Coates


### HEREDITARY HEMORRHAGE.

*Observations on Hereditary Hemorrhage.* By REYNELL COATES, M.D.†

THERE are several families, in various parts of the United States, affected with a singular predisposition to hemorrhage from very trivial injuries. Dr. OTTO, of this city, has written a paper, which will be found in the sixth volume of the New York Medical Repository, giving an account of this idiosyncrasy, as displayed in the descendants of a Mrs. Smith, who settled in the vicinity of Portsmouth, N. H., about eighty years previous to the date of his communication. Nearly all the male progeny of this lady were subject to dangerous, and often fatal, hemorrhage from the slightest wounds or scratches, nor could the most enlightened professional skill suggest any effective remedy, until it was accidentally discovered that the sulphate of soda exercised a control over these cases, as unaccountable as the affection itself.

The females of the family never exhibited this predisposition, although they transmitted it to their male issue. Those who were affected were robust men of a choleric disposition,

† North American Medical and Surgical Journal.

and many of their neighbours supposed that they could distinguish the "bleeders," as they were called, by certain peculiarities of personal appearance.

Another paper, by the same gentleman, published in Coxe's Medical Museum, vol. i., contains the particulars of the fate of four sons of Mr. Benjamin Binny, in Maryland; the first of whom lost his life by a hemorrhage, occasioned by the "kick of a horse over the eyebrow;" the second, from "the fall of a brick on the forefinger while at play; the third, from "a cut over the eye from the swing of a gate as he was passing through; and the fourth, who was subject to frequent attacks of epistaxis, fell a victim, in early life, to dropsy, consequent to the loss of blood. Physicians attended each of them, but to no purpose. The females of this family also were exempt from the affection.

Dr. Otto mentions that Dr. Rush was twice consulted in cases of the same hereditary tendency. Dr. Physick recently informed me that he had attended a patient, about thirty years ago, in whom a slight cut upon the knee produced a very obstinate hemorrhage, of a singular character; and I have lately been informed of two other families, one in Pennsylvania, and the other in Georgia, in which this idiosyncracy is strongly marked.

These instances are sufficiently numerous to render the history of the disease highly interesting to those practitioners who reside in the neighbourhoods where it prevails; and to such I hope the following case, in which I was recently consulted, will prove worthy of perusal. If the details of treatment should be considered unnecessarily minute, I must rest my apology upon the desire to avoid that careless and generalising style of description which renders too many reports of this character altogether useless to those who appeal directly to facts, and who are unwilling to place implicit confidence in the deductions of others.

R. W., a young gentleman from Delaware county, Pennsylvania, a member of the medical class, was the first of his family to display the idiosyncracy which is the subject of the present paper. His frame is vigorous, and he has every appearance of health.

His sister has never exhibited any uncommon disposition to hemorrhage, but among her children the following cases have occurred: A child, aged about eighteen months, fell, and ruptured the frœnum of the upper lip. Two of the most respectable practitioners of the neighbourhood exerted themselves to save the patient, but in vain: he bled to death.

Another child, aged about fifteen months, met with the same accident. Three physicians were consulted; but the hemorrhage continued until the third day after the injury, when the child was taken to Dr. Gideon Humphrey, in a state of extreme exhaustion. This gentleman succeeded in arresting the bleeding by means of the actual cautery.

The same child, when nearly thirteen years of age, received a cut upon the forehead by a blow from a stone, and was attended by two practitioners, who employed the actual cautery freely, but without effect: the patient fell a victim to the hemorrhage.

These cases are mentioned by Dr. Humphrey, in the Medical Review for September 1824. The slight discrepancy between the statements contained in that paper and the account here given, is owing to the fact that the former was published from rough notes, taken long after the event occurred, and not intended to meet the public eye; while the latter has been corrected by referring anew to Dr. H., and also to the family affected.

Dr. H. has since narrated to me two other cases in the same family. In the first case, which occurred in the brother of the children already mentioned, a slight contusion of the scrotum produced an enormous extravasation in the cellular tissue; and, in the second, a wound of the thumb was followed by bleeding for several days. The shower bath suddenly arrested the hemorrhage.

R. W., the gentleman above mentioned, received a cut from a penknife on the forefinger, when about eleven years of age. The wound continued to bleed without intermission; and on the fifth day after the accident Dr. Humphrey was consulted. He applied a compress and bandage "sufficiently tight to stop the discharge;" but on the following day he found the finger considerably inflamed, and the hand and forearm distended with extravasated blood. On removing the compress, the blood again flowed from the wound. The Doctor then applied remedies to combat the local inflammation, and continued them for a few days. The bleeding, however, did not cease, and the patient being in an extremely exhausted condition, the actual cautery was applied with complete success.

In the autumn of the same year, Mr. W. had a molar tooth extracted. Blood continued to ooze from the gum for many days; nor was the hemorrhage checked until the actual cautery was liberally employed as a last resort.

I shall now proceed to give the details of another similar attack, occurring to the same gentleman, when twenty-four years of age, as extracted from my case-book:

22d December, 1827, five o'clock P.M.—I was requested by Mr. (now Dr.) Spackman to visit Mr. W., who had a tooth extracted by a dentist the day before. The patient and his friends informed me that the hemorrhage following the operation was so profuse, and of so long continuance, that the dentist (at the request of Mr. W.) applied the actual cautery, but without success. Mr. Spackman took charge of the case at ten o'clock A.M.; but, being unable to check the discharge, he thought it advisable to call in additional aid.

The patient had lost at this time about half a gallon of blood. The measures resorted to by Mr. S. were as follows:

1st. The sulphate of copper was applied, in the solid form, to the bottom of the socket, without effect.

2d. A piece of cork, adapted to the form of the cavity, and wet with a strong solution of the same salt, was employed as a compress, with no better success.

3d. The cavity was plugged with lint, but without any benefit.

4th. A saturated solution of the sulphate of alumina was employed as a gargle; but this did not arrest the bleeding.

5th. A piece of sponge, imbued with this solution, was employed as a compress; a piece of cork was applied over it, and pressure was made by means of Barton's jaw bandage. This apparatus arrested the hemorrhage, but it returned in four hours.

6th. Dry sponge, covered with powdered sulphate of alumina, was applied, and secured in the same manner. This application lessened the amount of discharge considerably, but did not entirely check it.

At the time of my visit, I found Mr. W. weak and pale, with a pulse small and frequent, but quite mild. His stomach was irritable. The bleeding at this time being slight, I did not think proper to advise the removal of the dressings; but recommended that the patient should be kept cool and quiet, in the hope that the hemorrhage might cease, without obliging us to resort to more severe measures.

23d December.—I was called to Mr. W. in haste, at ten o'clock A.M., and found him bleeding profusely. The dressings were removed; and, in order finally to test the effect of pressure applied in the most careful manner, I proceeded to plug the cavity of the socket with dry lint, firmly impacted by means of a bent wire. Over this was placed a firm compress of lint, surmounted by a piece of cork, upon which the inferior jaw was strongly closed, by means of Barton's bandage. This apparatus totally suppressed the discharge until after daylight, when it returned.

At ten o'clock A.M. the hemorrhage was very considerable. I reapplied the compresses, moistened with a saturated solution of sulphate of copper. The bleeding was rather less profuse for half an hour; but after this time it became even more copious than I had yet seen it.

At eleven o'clock, Mr. Spackman being present, we made a very careful examination of the wound, and discovered that no blood escaped from the socket of the tooth; but, on the contrary, that the discharge took place from one angle of the incision made by the gum lancet, just within the tooth next adjoining, which was the first of the molares. The whole cheek and gum were much swelled and inflamed, in consequence of the pressure which had been employed; and there was some febrile action. The actual cautery was now applied to the bleeding surface, and the hemorrhage became instantly arrested. The patient was ordered to take a purge of the sulphate of magnesia immediately; to be followed by the acetate of lead and the nitrous powder,* alternately exhibited.

At the request of the patient, Dr. Gideon Humphrey was called in consultation.

At three o'clock P.M. Dr. H. met us. The hemorrhage was very slight, and no change of measures was proposed. Dr. H. stated that, in all the cases occurring in this family, pressure invariably occasioned severe inflammation, without producing any benefit.

At eleven P.M. I found the hemorrhage as profuse as ever. I reapplied the cautery freely, and checked the discharge.

26th December.—I did not see the patient on the 24th or 25th, concluding that, as I had not been sent for, the case had terminated favorably. Mr. Spackman, however, informed me that the hemorrhage had recurred on the morning of the 26th, and continued without intermission, notwithstanding the application of almost every styptic which suggested itself to Mr. W. and his attendants. Most of the remedies appeared to lessen the flow for a short interval, but their ulterior effect was rather to increase it.

At ten o'clock this morning I called, and found the patient absent. Weak as he was, he walked to Dr. H.'s office, where the cautery was again applied very freely to the inner flap, the bleeding being confined to that part only. The discharge was again checked, but returned as violently as ever on his arrival at his own residence. The cautery was frequently reapplied by myself and others during the day and night, but without permanent advantage.

27th December.—The consultation met at ten o'clock A.M. The local inflammation had in a great degree subsided. Mr. Spackman stated to us that, at one A.M., Mr. W. had been attacked with vomiting, purging, and considerable pain about the colon. With a view to relieve these symptoms, Mr. S. exhibited Calomel grs. iij. cum Pulv. Opii gr. j., and directed Tinct. Opii gtt. xxx. quâque tertiâ horâ sumend.

The patient was now free from fever, and the purging had ceased, but the stomach continued irritable. This symptom was relieved by a spice plaster to the epigastrium, and a Seidlitz powder, which operated freely. The hemorrhage was very profuse, and the patient extremely exhausted.—Tinct. Opii continued.

* The nitrous powder contains Nitr. Potass. grs. x. with Tart. Antimon. gr. 1-6th. It is a formula employed in the practice of the Pennsylvania Hospital.

It was resolved today to make a final conclusive trial of the cautery, under the idea that some portion of the flap might have escaped contact with the iron, I therefore proceeded to destroy, by this means, not only the wounded surface, but the whole thickness of the gum and surface of the socket. The blood continued to ooze rapidly through the substance of the slough, and, about an hour after the application of the iron, I distinctly saw the hemorrhage proceeding from the surface of the uninjured gum, at some distance from the sloughs. This exudation continued for a short-time only.

At the request of the friends of the patient, Dr. J. R. Barton was invited to join us in consultation.

At two p.m. the consultation met. Hemorrhage considerable. The blood contained very little fibrin. The patient was exceedingly pale, and his pulse very weak, but perfectly mild. The amount of discharge since the commencement of the case certainly exceeds two gallons. It was agreed that a nourishing diet had become indispensable: he was, therefore, ordered to take plentifully of soups and jellies; eggs were also allowed him. Port wine was directed to be given pretty freely; and the spirit of turpentine was prescribed, fifteen drops to be taken every two hours.

At six p.m. I called. Mr. W. had taken about half a pint of port wine. The spirit of turpentine was immediately rejected from the stomach. The patient had considerable fever, with some stupor and pain in the head. The port wine and turpentine were therefore omitted, and gruels and beaten eggs directed to be taken freely: mustard plasters were applied to the feet. During the following night the hemorrhage declined, and ceased entirely for an hour after a warm breakfast.

28th December.—At ten a.m. the consultation met D r. Humphrey absent. The patient was free from fever, and the bleeding not very profuse. The exhibition of port wine was renewed, and Huxham's tincture of bark was prescribed.

Twelve p.m.—Found considerable fever, the pulse beating 122 strokes in a minute, and the hemorrhage profuse. Applied the muriated tincture of iron, and, at the earnest desire of the friends, permitted the application of a popular remedy, the oil obtained from burnt linen. Both these articles lessened the discharge for a short time, but ultimately produced an exacerbation. The compound tincture of bark being invariably rejected, its exhibition was discontinued.

29th December.—At eleven a.m. the consultation met. Mr. W. informed us that the hemorrhage had not abated until he had taken breakfast, after which it had stopped for half an hour. At the time of our visit it was considerable. The fever had somewhat abated; pulse 115. Notwithstanding the free purging which followed the exhibition of calomel two days before, a strong mercurial odour was perceptible in the patient's breath.

Ordered Sulph. Quiniæ gr. j. q. tertiâ hor. sum., et Tinct. Ferri gtt. v. q. hor. sum.—Tinct. Opii continued.

At the desire of the friends of the patient, Dr. Physick was requested to join us in consultation.

At four p.m. Dr. P. and myself met, the other gentlemen being absent. One important indication in the treatment of hemorrhages from the mouth in general was suggested by Dr. P. He stated that he had met with instances of extreme exhaustion from bleeding, consequent to operations in that cavity, and which continued after all the remedies usually resorted to had been employed to no purpose. These discharges he supposed might be kept up by the suction unavoidably attendant upon the effort to swallow, and were to be arrested by keeping the mouth permanently open.

In order to answer this indication as completely as possible in Mr. W.'s case, a plug of wood was introduced between the molar teeth, and he was ordered to receive nourishment through the stomach tube. He was also confined to a sitting posture; and, in order to ascertain whether the coagulation of the blood upon its exit would not prove serviceable, when aided by the means just mentioned, it was directed that a slight pledgit of common spunk should be kept in contact with the bleeding surface.

The introduction of the stomach tube was strongly resisted by the patient, under the impression that some injury might be sustained by the œsophagus, in which a second hemorrhage, less accessible, and consequently more unmanageable, might result.

At one p.m. the bleeding continued unchecked, notwithstanding the introduction of the plug, which the patient was compelled to remove in about an hour, in consequence of the nausea occasioned by its presence. He took one egg beaten up with sugar and water, and this was his only sustenance for twenty-four hours.

At eleven p.m. I reapplied the plug, but the extreme irritability of the fauces rendered its presence intolerable, and Mr. W. removed it at one o'clock a.m. He then continued to hold spirit of turpentine in his mouth for some hours, at his own suggestion, but this increased the hemorrhage very considerably.

Dec. 30th.—At ten a.m. we met. We were informed that the patient had taken plentifully of warm tea for breakfast, after which the hemorrhage was much diminished for some time. The mercurial odour could no longer be detected in the breath.

At four p.m. Mr. Spackman, having procured some spunk, introduced a wedge-shaped plug between the molares of the injured side, surmounted by a pledgit of that article, according to the directions of yesterday. The hemorrhage, although somewhat lessened for a short interval, ultimately became still more violent. I afterwards carefully reapplied the same apparatus, with a similar result.

The patient was soon afterwards attacked with vomiting and purging. Mr. Spackman exhibited laudanum freely, and these symptoms were soon relieved. He then reapplied the plug, with temporary benefit; but Mr. (now Dr.) TEMPLE, in whose charge the case was placed for the night, states that the bleeding became very profuse in a short time. At three o'clock A.M. efforts to vomit rendered it necessary to remove the plug. The patient was now extremely weak, having nearly fainted on attempting to rise. After the final removal of all local applications, the hemorrhage ceased in a little while, and did not again return until five P.M.

Dec. 31st.—At ten A.M. we again met, all the gentlemen being present. The patient had taken plentifully of rice broths, rice pudding, and other nutritious articles not requiring mastication. This diet was ordered to be continued. He was directed to prevent the entire closure of the teeth, but not of the lips, by inserting a small piece of cork between the incisors. The amount of Tinct. Opii was reduced to gtt. xxx. every four hours. At five P.M. the hemorrhage returned, after a slight febrile paroxysm, accompanied by chillness and headache. It continued to flow gently until three o'clock in the morning.

1st January, 1828.—In the afternoon of this day there was a slight return of bleeding, which continued two hours, and then finally ceased. The whole amount of blood lost during the progress of the case cannot be estimated at less than three gallons.

It is proper here to remark that Mr. W. has received several wounds from the sickle, while engaged upon his father's farm. One of these, which was a severe and deep cut upon the hand, produced no unusual discharge of blood; but several others, of less extent, required the application of the actual cautery. The slight scratches occasionally inflicted by the razor seldom caused any trouble.

(To be continued.)

---

